# Effectiveness of the “Living with Cancer” peer self-management support program for persons with advanced cancer and their relatives: study protocol of a non-randomized stepped wedge study

**DOI:** 10.1186/s12904-022-00994-5

**Published:** 2022-06-13

**Authors:** K. L. Luu, F. E. Witkamp, D. Nieboer, E. M. Bakker, L. W. Kranenburg, C. C. D. van der Rijt, K. Lorig, A. van der Heide, J. A. C. Rietjens

**Affiliations:** 1 Department of Public Health, Erasmus MC, University Medical Center Rotterdam, Rotterdam, the Netherlands; 2 Center of Expertise Innovations in Care, Rotterdam University of Applied Sciences, Rotterdam, the Netherlands; 3 Department of Psychiatry, Section Medical Psychology and Psychotherapy, Erasmus MC, University Medical Center Rotterdam, Rotterdam, the Netherlands; 4 Department of Medical Oncology, Erasmus MC, University Medical Center Rotterdam, Rotterdam, the Netherlands; 5 Stanford School of Medicine, CA Stanford, USA

**Keywords:** Self-management program, Peer support, Peer-led, Advanced Cancer, Relatives, Video-conferencing, Non-randomized stepped wedge

## Abstract

**Background:**

Persons with advanced cancer and their relatives experience physical, emotional, and psychosocial consequences of the illness. Most of the time, they must deal with these themselves. While peer self-management support programs may be helpful, there is little evidence on their value for this population. We present the research protocol of our SMART study that will evaluate the effectiveness of the “Living with Cancer” peer self-management support program, aimed at improving self-management behaviors, self-efficacy, and health-related quality of life of persons with advanced cancer and their relatives.

**Methods:**

We will conduct a non-randomized stepped wedge study in the Netherlands. We will include 130 persons with advanced cancer and 32 relatives. Participants can choose to either start the program within 4 weeks after inclusion or after eight to 10 weeks. The “Living with Cancer” is a peer self-management support program, based on the Chronic Disease Self-Management Program. It consists of six 1,5 hours video-conferencing group meetings with eight to 12 participants, preceded by two or three preparatory audio clips with supportive text per session. The program has the following core components: the learning of self-management skills (action-planning, problem-solving, effective communication, and decision-making), discussing relevant themes (e.g. dealing with pain and fatigue, living with uncertainty, and future planning), and sharing experiences, knowledge, and best practices. The primary outcome for both persons with advanced cancer and relatives is self-management behavior assessed by the subscale “constructive attitudes and approaches” of the Health Education Impact Questionnaire. Secondary outcomes are other self-management behaviors, self-efficacy, health-related quality of life, symptoms, depression and anxiety, and loneliness. Participants complete an online questionnaire at baseline, and after eight and 16 weeks. After each session, they complete a logbook about their experiences. Group meetings will be video recorded.

**Discussion:**

SMART aims to evaluate an innovative program building on an evidence-based self-management program. New features are its use for persons with advanced cancer, the inclusion of relatives, and the video-conferencing format for this population. The use of both quantitative and qualitative analyses will provide valuable insight into the effectiveness and value of this program.

**Trial registration:**

This study was registered in the Dutch Trial Register on October 2021, identifier NL9806.

**Supplementary Information:**

The online version contains supplementary material available at 10.1186/s12904-022-00994-5.

## Background

In recent years, there has been a shift towards participatory healthcare [1, 2]. Persons with advanced cancer are increasingly expected to take up more responsibility for their health and care at home. Together with their relatives, they have to deal with physical, emotional, psychosocial, complex treatment regimens, and lifestyle consequences of the illness [3, 4]. Due to new treatments, several cancer types are evolving towards a chronic condition, and many patients live with their illness for years [4, 5]. As a result, persons with advanced cancer and their relatives are faced with long term uncertainties, for instance, whether the illness will progress or how they should best deal with the illness [6]. In addition, relatives have a high risk of ‘caregiver burden’ [7]. They can sometimes be in the conflicting position of both giving support to the person with advanced cancer, while also having to deal with the consequences of advanced cancer in their own lives [8, 9].

Self-management is about how a person with a long term condition deals with medical, role, and emotional issues [10]. A key factor in improving self-management is self-efficacy, which refers to people’s beliefs in their capabilities to perform specific behavior [10]. Self-management of persons with an advanced illness has been defined as “the strategies to manage the physical, psychosocial and existential consequences of living with a progressive, life-threatening disease and its treatment.” [11]. Key to this definition is that self-management involves more than symptom management alone and includes the management of problems in other domains, such as psychosocial problems [11]. Therefore, self-management support interventions ideally include both persons with long term conditions and their relatives [9]. Most self-management studies are developed for persons with chronic diseases and are found to reduce the severity of symptoms and improve quality of life [12–14].

One way to support self-management and enhance self-efficacy is through peer support and modeling. This is the support of persons who share their experiential knowledge, and/or emotional, social or practical support to others in similar conditions [15]. Peer support can be provided one-to-one or in a group, it can be professional-led or peer-led, and it can be delivered face-to-face or online [16]. Online peer support can be asynchronous (such as online discussion boards) or synchronous (through video-conferencing). For persons with cancer, a recent review indicates that peer-led peer support has positive effects on coping, self-efficacy and cancer-related knowledge, regardless of the mode, duration and format of the intervention [16]. Most studies in this review were conducted among persons with breast cancer [16]. Despite its potential value, few studies have been conducted addressing the effectiveness of peer support among persons with advanced cancer or their relatives [17–19].

After examining several programs, we identified the Chronic Disease Self-Management Program (CDSMP), developed in the USA by Lorig et al. [20], as among the most effective and potentially relevant given its use in diverse populations, in different countries and cultures, and its peer-led format [12, 13]. A meta-analysis of 23 studies of the CDSMP demonstrated improved self-efficacy, health behaviors (such as exercise, cognitive symptom management and communication with physicians) and physical health outcomes (energy, shortness of breath, fatigue, pain and self-rated health) [13]. The original CDSMP has several adapted versions for various populations [21]. We developed an adapted version of the CDSMP to meet the needs of persons with advanced cancer and their relatives in the context of the Netherlands. Given the increasing symptom burden, limited energy of this population, and the Covid-19 pandemic, we have chosen to deliver the program via video-conferencing [22].

In this article, we present the research protocol of our SMART study to evaluate the “Living with Cancer” program for persons with advanced cancer and their relatives. We used The Standard Protocol Items: Recommendations for Interventional Trials (SPIRIT) to describe relevant aspects of the study [23]. See Additional file 2 for the SPIRIT checklist.

### Objectives

The overall aim of this study is to assess the effectiveness of the program on self-management behaviors, self-efficacy and health-related quality of life of persons with advanced cancer, and explore its effectiveness in a smaller group of relatives. Moreover, through qualitative analyses, we will study the experiences of both groups with the program.

## Methods

### Intervention

#### Development intervention

The “Living with Cancer” program is a peer self-management support program based on self-efficacy theory [20, 24]. The program was built on two adapted versions of the CDSMP, the “Cancer Thriving and Surviving” program (developed for persons affected by cancer) and the “Building Better Caregivers” program (developed for caregivers of persons with cognitive impairments) [25, 26]. The core of these programs was preserved and some of the content was replaced with relevant themes for our specific group. In close collaboration with the director of the CDSMP (K. Lorig), changes were made based on findings from our systematic review [27] and in-depth interview studies [28, 29], see Additional file 3 for related articles. The content of the “Living with Cancer” program was discussed in reference groups of patients and relatives (*N* = 10), healthcare professionals (*N* = 5) and researchers in palliative care (*N* = 8). A pilot study was conducted to evaluate the initial acceptability and feasibility of the “Living with Cancer” program. In this pilot, 12 participants (seven persons with advanced cancer, three relatives and two potential peer facilitators) completed the program and an online questionnaire (see Table 4). They were interviewed about their experiences. Participants evaluated the program as acceptable and feasible, with a mean satisfaction score of 8.5 on a scale of 0–10.

#### Content and format

The “Living with Cancer” program consists of two parts: six 1,5 hours video-conferencing group meetings with eight to 12 participants, facilitated by two peers, and preparatory audio clips. The latter concerns 15 three-minute preparatory audio clips with supportive text, addressing essential information about the themes that will be discussed in the meetings. As an additional resource, participants receive a workbook containing relevant chapters from the CDSMP book “Living a healthy life with chronic conditions” [44] and links to evidence-based information related to the themes in the program.

The program will be delivered by “Zoom” video-conferencing. To prepare and support participants for the meetings, there is a brief “meeting zero” with every individual participant to test the technical procedures. Technical support is available during the six video-conferencing group meetings.

The meetings focus on the development of participants’ self-management skills: action-planning, problem-solving, effective communication and decision-making [10]. In the meetings, these skills are related to relevant themes. Table 1 provides an overview of the program.

**Table 1:** Overview of the “Living with Cancer” program

Meeting 1	Meeting 2	Meeting 3	Meeting 4	Meeting 5	Meeting 6
Introduction programme	Sharing experiences; Action plan; Problem solving	Sharing experiences; Action plan	Sharing experiences; Action plan	Sharing experiences; Action plan	Sharing experiences; Action plan
Introduction participants	Improving communication Listening activity	Problem solving Sharing activity	Decision making Sharing activity	Communication with ourselves Sharing activity	Adapting lifestyle Brainstorm
Mind body connection Breathing exercise	Dealing with difficult emotions Brainstorm	Living with uncertainty Brainstorm and sharing activity	Planning the future Brainstorm	Dealing with pain Brainstorm	Intimacy/ Sexuality Brainstorm
Dealing with fatigue and prioritizing Brainstorm	Formulation action plans	Guided imagery Listening activity	Communication with healthcare professionals Brainstorm	Improving communication with family Brainstorm	Reconnecting to people and getting help Brainstorm
Introduction to action plans	Instructions preparation next meeting	Formulation action plans	Formulation action plans	Formulation action plans	Evaluation
Instructions preparation next meeting		Instructions preparation next meeting	Instructions preparation next meeting	Instructions preparation next meeting	

The group meetings are structured. The facilitators introduce the activities and themes. After giving information, they initiate conversations between participants by guiding brainstorms and sharing experiences. They lead exercises (e.g. breathing) and listening activities (e.g. relaxation). The facilitators support participants in selecting challenges on which they would like to work, but do not suggest activities (this is called self-tailoring), and encourage them in completing their self-selected goals [20]. For detailed information about the program, see the Template for Intervention Description and Replication Checklist in Additional file 1 [45].

#### Peer facilitators

Facilitators are peers and are persons with stable (advanced) cancer, cancer survivors, relatives of persons with cancer or bereaved relatives of patients who died at least 6 months before the facilitators’ training. Peer facilitators are trained to follow a structured protocol with standardized scripts, to ensure consistency of delivery and content of the program. This requires a 24-hour online training distributed over 6 weeks, in which they participate in the program and learn how to facilitate it. Two certified master trainers provide the training, consisting of content delivery, adherence to timing and sequence of themes, coverage of the activities as set out in the protocol, and dealing with sensitivities and specific complex situations. Peer facilitators are instructed that they are not allowed to give medical advice. To ensure the quality of the meetings and adequate delivery of the content, fidelity will be checked with the CDSMP fidelity checklist by a selection of the recorded meetings.

### Study design

**Fig. 1:**
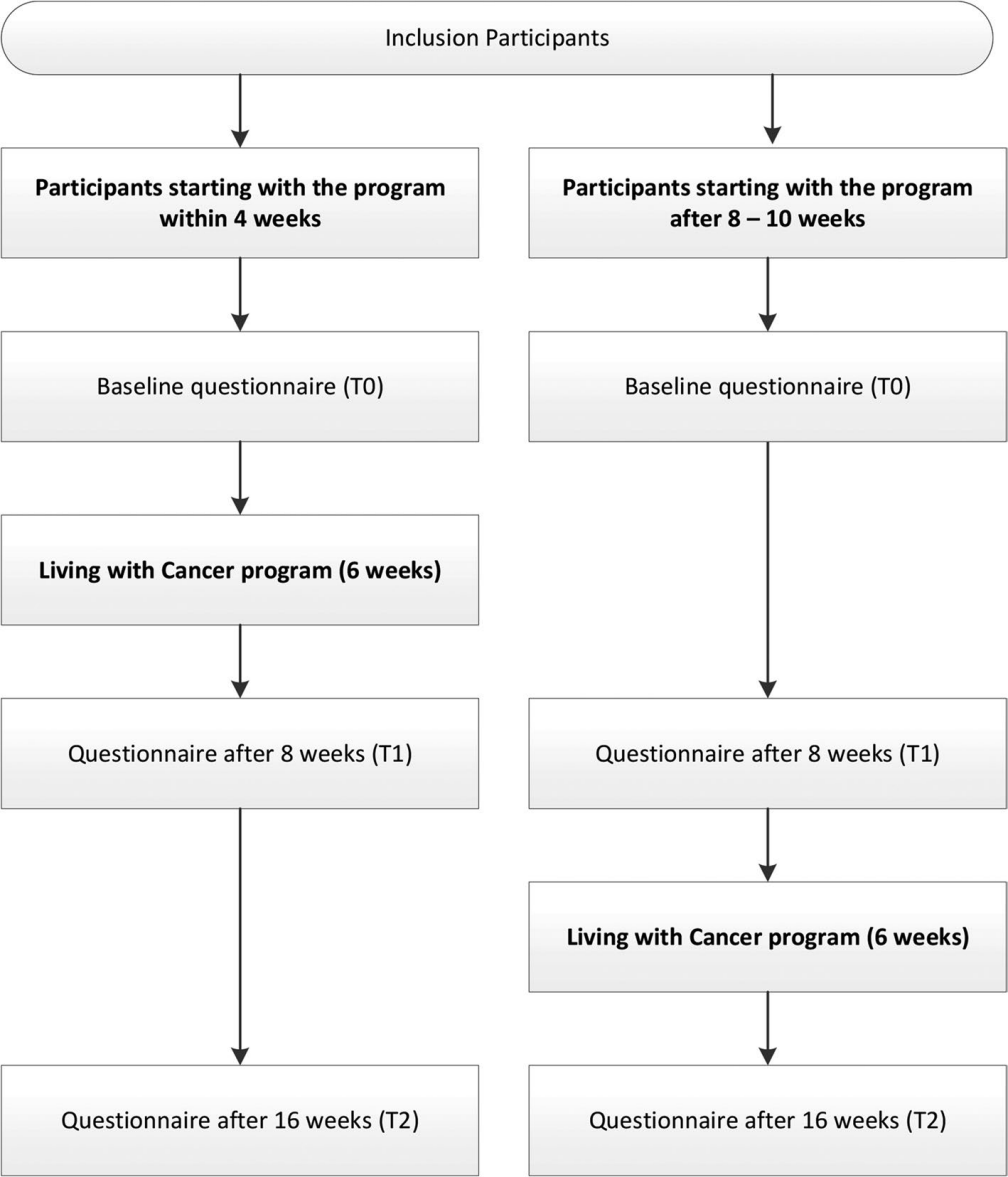
Overview of the non-randomized stepped wedge design

We will conduct a non-randomized stepped wedge study [46]. Participants can choose to either start the program within 4 weeks after inclusion (early starters) or after 8 to 10 weeks (late starters). The late starters allow us to collect control data about participants who have not yet followed the program [47]. See Fig. 1 for an overview of the non-randomized stepped wedge design.

### Study population

Patients and relatives can participate together in the study, in the same or separate groups, as preferred. Patients can also participate without a relative and vice versa. Relatives can be partners, parents, children and other significant others such as close friends or neighbors.

Inclusion and exclusion criteria are described in Table 2 for patients and Table 3 for relatives.

**Table 2:** patients’ inclusion and exclusion criteria

Inclusion criteria	Exclusion criteria
Having advanced cancer (defined as having no curatively aimed treatment options available, only life-prolonging or palliative treatments)	Younger than 18 years of age
Conform WHO performance status of 0 or 1 (34)	Unable to provide written informed consent
Able to read and speak the Dutch language	Not willing to use a camera
Access to a computer or laptop and internet

**Table 3:** relatives’ inclusion and exclusion criteria

Inclusion criteria	Exclusion criteria
Relative of a patient with advanced cancer	Younger than 18 years of age
Able to read and speak the Dutch language	Unable to provide written informed consent
Access to a computer or laptop and internet	Not willing to use a camera

### Recruitment

Participants will be recruited in two ways: via participating hospitals (an academic cancer center and a general hospital) and via self-referral. Self-referral has been described to increase ecological validity, facilitate greater equity of access to psycho-oncology research, and facilitate faster implementation of effective interventions into clinical practice [48]. The study will be advertised through diverse online channels such as a Dutch cancer information website [49], social media (Twitter, LinkedIn and Facebook), newspapers, patient associations and our website: www.smart-onderzoek.nl [50]. Persons with advanced cancer will be asked to invite one of their relatives to participate and vice versa.

Participants will receive the participant information sheet and oral information. They will be given adequate time (at least 1 week) to read and consider participation. Written consent will be obtained without any coercion of participants.

### Measurements

In the SMART study, we will take the following measurements:

- Questionnaire study. All participants will fill in an online questionnaire at three moments: right after inclusion (baseline), after 8 weeks and after 16 weeks. Completion of a questionnaire will take approximately 20 minutes. A written questionnaire can be provided if preferred. We will use both validated and self-constructed questionnaires (for an overview of the measures, see Table 4).
- Logbooks. After each meeting, participants will be asked to answer a set of questions in a logbook: What did you learn during the meeting? In what way was the meeting useful (if at all)? How was the group experience? Which exercises were useful? Participants will be asked to document their action plans.
- Recorded video-conferencing sessions. The group meetings will be video recorded with the consent of the participants.

**Table 4:** Measurements tools

Measurement tools	
Concept	Measured by
Primary outcome:	
Self-management behavior	Health Education Impact Questionnaire (HEIQ) [30] **Patients and relatives:** Subscale ‘constructive attitudes and approaches’ (*5 items)*
Secondary outcomes:	
Self-management behaviors	Health Education Impact Questionnaire (HEIQ) [30] **Patients:** Subscales ‘skill and technique acquisition’ (*4 items)*, ‘health services navigation’ (*5 items)*, ‘social integration and support’ (*5 items)* **Relatives:** Subscales ‘health directed behavior’ (*4 items)*, ‘positive and active engagement in life’ (*5 items)*, ‘social integration and support’ (*5 items)*
Quality of life	**Patients:** McGill Quality of Life (MQOL) [31, 32] (*18 items)* **Relatives:** Quality of Life in Life Threatening Illness Family Carer Version (QOLLTI-F) [33] Subscale ‘overall quality of life’ (*1 item)*
Self-efficacy	**Patients:** Self-efficacy for managing chronic disease [24] Adaptation to advanced cancer *(6 items)* **Relatives:** Self-efficacy caregivers short adaption [34] Adaptation to advanced cancer (*7 items)*
Symptoms	**Patients and relatives:** Fatigue numeric rating scale (NRS) (*1 item)* **Patients:** Pain numeric rating scale (NRS) (*1 item)* **Relatives:** Stress numeric rating scale (NRS) (*1 item),* Sleep numeric rating scale (NRS) (*1 item)*
Caregiver burden	**Relatives:** Caregiver Reaction Assessment Dutch (CRA-D) [35]; Subscale ‘impact on schedule’ (*5 items)*
Depression and Anxiety	**Patients and relatives:** Hospital Anxiety and Depression (HADS) [36] *(14 items)*
Loneliness	**Patients and relatives:** University of California, Los Angeles (UCLA) loneliness scale [37] Short version *(3 items)*
Other measures:	
Healthcare utilization	**Patients and relatives:** Number of contacts with healthcare professionals, number of hospitalization days, reasons for hospitalization, number of visits to accident and emergency departments [38] Self-constructed (*6 items)*
Sociodemographic characteristics	**Patients and relatives:** Age, gender, ethnicity, Social Economic Status (SES), marital status, cancer type and time since diagnosis Self-constructed (*15 items)* **Relatives:** Hours of caregiving per week *(1 item)*
Comorbidity	**Patients and relatives:** Self-Administered Comorbidity Questionnaire (SCQ) [39] *(1 item)*
Health literacy	**Patients and relatives:** Degree of understanding medical information, Set of Brief Screening Questions (SBSQ) [40] *(3 items)*
Resilience	**Patients and relatives:** Connor-Davidson Resilience Scale (CD-RISC) [41] *(10 items)*
Digital comfort and skills	**Patients and relatives:** The comfort and skills of using computer and mobile devices [42] (*2 items)*
Group cohesion	**Patients and relatives:** The Group Climate Questionnaire (GCQ-23) [43] *(23 items)*
Evaluation of the program	**Patients and relatives:** Experiences with the program Self-constructed (*7 items)*

### Sample size calculation

To show an effect size of at least 0.5 SD on the HEIQ scale “constructive attitudes and approaches” [51], with a power of 80% and a significance of 0.05, assuming an individual autocorrelation across different time points of 0.7 [51], and assuming an average cluster size of eight patients simulation showed that in total 104 patients would need to be included across a wide range of within-period ICCs. With an expected drop-out rate of 20% (similar to the various CDSMP programs [24–26]), we need to recruit at least 130 patients. Based on our pilot study, we expect that of the participants, 20% will be relatives, therefore, we expect to include at least 32 relatives. In total, we will include 162 participants.

### Data management

We will use the online survey tool Lime Survey [52] to send all questionnaires to participants. Gems Tracker will be used to uniformly collect, store and analyze the data [53]. Recordings of the meetings and logbooks will be stored at the Erasmus MC, University Medical Center Rotterdam. Only the research team will have access to the data.

### Statistical analysis

We will follow the intention-to-treat principle for the analyses of the primary and secondary outcomes. Descriptive statistics will be used to summarize patient characteristics (age, gender, ethnicity, Social Economic Status, marital status, cancer type and time since diagnosis).

For the patient outcomes, we will use linear mixed models with a random intercept to adjust for repeated measures over time. As fixed effects, we will include time points of measurement (baseline, 8 weeks and 16 weeks) and a variable denoting when the participant received the program at each time point (early versus late starters). To adjust for possible confounding, we additionally will add age, sex, ethnicity, Social Economic Status, cancer type and time since diagnosis as fixed effects to the model.

Furthermore, we will assess whether there is a dose-response relationship between the number of sessions of the program and outcome. We will perform a similar analysis as for the main effect, but include the number of sessions instead of the intervention group.

The quantitative data analyses for relatives will be conducted in a similar way but it will have an explorative nature due to its expected lower number of participants.

### Qualitative research

A complementary qualitative study will be carried out to explore the lived experiences of participants with the “Living with Cancer” program. Data concern the recordings of the video-conferencing groups and participants’ logbooks. We will conduct inductive, thematic content analyses [54, 55] describing their experiences with the program, including their perceived value of group-based peer support, the video-conferencing format, and the approach of including both persons with advanced cancer and relatives. We will also explore the perceived working mechanism of the program.

## Discussion

Self-management for persons with advanced cancer and their relatives is multifaceted due to multilayered consequences and the uncertainties they face. To our knowledge, there is a lack of research into peer self-management support for this population.

The SMART study aims to fill this gap in knowledge. We will evaluate a self-management program to support persons with advanced cancer and their relatives. The program has been built on the CDSMP, an evidenced-based peer self-management support program for persons with chronic conditions [12, 13]. New features are its use for persons with advanced cancer, the inclusion of relatives, and the video-conferencing format for this population. The use of both quantitative and qualitative analyses will provide valuable insight into the effectiveness and value of this program.

Peer support for persons with advanced cancer and their relatives has rarely been researched [17]. Although the concept of peer support dates back several centuries, it is only in the last few decades that it has tracked attention in healthcare. In the field of mental health, there is an international growing trend to adopt peer support [56]. The literature suggests that peer support is beneficial in mental health care, such as improved health-related quality of life and improved patient activation [56]. In this study, we will now explore its value, as part of a self-management support program for persons with advanced cancer and their relatives. Self-management programs for this population with a format of video-conferencing group meetings are also new. A review showed that group interventions delivered by video-conferencing are acceptable and feasible in various populations (such as chronic disease, obesity, caregivers) [22]. In healthcare, telemedicine can be of considerable benefit to patients [57], and induced by the Covid-19 pandemic, it has taken off [58]. Therefore, examining this format for persons with advanced cancer and their relatives is a relevant next step.

Our SMART study has several risks. The study population and the program contain several factors that cannot be fully controlled. Firstly, there will be a considerable risk of drop out, either partly (some meetings) or completely, to disease progression or treatment burden of our vulnerable population [59]. To minimize this, we only include patients with a WHO performance status of 0 or 1 [60], and participants can choose when to start with the program to accommodate their schedule. Secondly, the risk of selection bias cannot be ruled out. Conducting research through self-referral, by online questionnaires and the video-conferencing group format, may primarily attract participants with higher digital literacy [61]. To minimize this risk, we offer technical support before and during the program, and explicitly explain that experience with video-conferencing applications is not necessary. Banbury and colleagues demonstrate that inexperience with video-conferencing or computer use was not a major problem for participation in video-conferencing groups [22]. Thirdly, while a randomized controlled trial (RCT) is preferred as a gold standard for measuring the effects of an intervention, we opt for a non-randomized stepped wedge design to make the study better feasible. Whilst we adjust for possible confounders in the analysis, residual confounding still remains a risk. Fourth, our program is a complex intervention, meaning it contains several interacting components, such as improving self-efficacy and behavior, the self-tailoring format, and group interaction [62]. Therefore, it may be difficult to identify the active components of the program. Combining the quantitative data of this study with the findings of our complementary qualitative study will provide insight into participants’ experiences and the perceived working mechanism of the program.

## Supplementary Information

**Additional file 1.**

**Additional file 2.**

**Additional file 3.**

## Data Availability

The datasets used and/or analysed during the current study are available from the corresponding author on reasonable request.

## Abbreviations

CDSMP: Chronic Disease Self-Management Program
CD-RISC: Connor-Davidson Resilience Scale
CRA-D: Caregiver Reaction Assessment Dutch
GCQ-23: The Group Climate Questionnaire
HADS: Hospital Anxiety and Depression
HEIQ: Health Education Impact Questionnaire
ICC: intraclass correlation coefficient
MQOL: McGill Quality of Life
NRS: numeric rating scale
QOLLTI-F: Quality of Life in Life Threatening Illness Family Carer Version
RCT: Randomized Controlled Trial
SBSQ: Set of Brief Screening Questions
SCQ: Self-Administered Comorbidity Questionnaire
SES: Social Economic Status
SPIRIT: Protocol Items: Recommendations for Interventional Trials
UCLA: University of California, Los Angeles
WHO: World Health Organization
