# Supplementary material for: Effectiveness of the “Living with Cancer” peer self-management support program for persons with advanced cancer and their relatives: study protocol of a non-randomized stepped wedge study
Source: BMC Palliat Care. 2022 Jun 13;21:107. doi: 10.1186/s12904-022-00994-5 (PMC9188837; doi:10.1186/s12904-022-00994-5)
Supplement: Supplementary file 1 — Additional file 1. [file 12904_2022_994_MOESM1_ESM.docx]

**Summary of the “Living with Cancer” program using the Template for intervention description and replication (TIDieR) checklist**

| **TIDieR number** | **TIDieR item** | **“Living with Cancer” program** |
| --- | --- | --- |
| 1 | BRIEF NAME (name or a phrase that describes the intervention) | The “Living with Cancer” program: a peer-led, peer-support, video-conference group program promoting self-management of persons with advanced cancer and their relatives. |
| 2 | WHY (any rationale, theory, or goal of the elements essential to the intervention) | Self-management has a broad definition and involves medical management, role management and emotional management of living with the disease.  The "Living with Cancer" program, based on the Chronic Disease Self-Management Program (CDSMP), has a broad approach and is based on self-efficacy theory.  The focus is to develop or improve participants’ self-management skills that will allow them to better deal with the physical, psychosocial and practical problems that they may encounter as a consequence of their or their relative’s illness and/or treatment. |
| 3 | WHAT Materials (any physical or informational materials used in the intervention, including those provided to participants or used in intervention delivery or in training of intervention providers) | The core of the “Living with Cancer” program consists of six 1,5 hours video-conferencing group meetings, each combined with short preparatory audio clips. Furthermore, there are some additional resources.  Seven intervention materials are provided to support the program:  1. “Zoom”. A video-conferencing application to deliver the program.  2. 15 three-minute preparatory audio clips with supported text. Participants prepare themselves for the upcoming meetings by listening and watching these audio clips.  3. Workbook. This is an additional supporting resource with chapters from the CDSMP book “Living a healthy life with chronic conditions” and links to evidence-based information related to the themes in the program.  4. Facilitator’s manual. A protocol for facilitators on how to deliver the program.  5. PowerPoints. Facilitators use PowerPoints to show the agenda of the meeting, charts and tables.  6. Mastertrainer’s manual. A protocol for Master trainers to train facilitators.  7. PowerPoints. Master trainers use PowerPoints to show the agenda of the training and demonstrate scenarios, charts, and tables. |
| 4 | WHAT Procedures (each of the procedures, activities, and/or processes used in the intervention, including any enabling or support activities) | Six 1,5 hours video-conferencing group meetings.   - Meeting 1: The first meeting begins with welcoming the participants and outlining the content and process of the program. Topics covered include “Mind-body connection” with a breathing exercise, “Dealing with fatigue and prioritizing”, and “Introduction Action plans”. - Meeting 2: Topics covered include “Improving communication with a listening activity”, “Dealing with difficult emotions” and “Action plans”. - Meeting 3: Topics covered include “Problem solving”, “Living with uncertainty”, “Guided imagery” and “Action plans”. - Meeting 4: Topics covered include “Decision making”, “Planning the future”, “Communication with healthcare professionals” and “Action plans”. - Meeting 5: Topics covered include “Communication with ourselves”, “Dealing with pain”, “Improving communication with family and friends” and “Action plans”. - Meeting 6: Topics covered include “Adapting lifestyle”, “Intimacy/Sexuality”, “Reconnecting to people and getting help”, and “Evaluation”.   Fifteen preparatory audio clips.  Audio clips 1 to 14 address essential information about the themes that will be discussed in the meetings. They serve as an introduction to the themes and discussions. Audio clip 15 is an optional guided imagery script. |
| 5 | WHO PROVIDED (intervention provider, their expertise, background and any specific training given) | The meetings will be facilitated by peers (called ‘facilitators’) who completed the facilitators’ training of the “Living with Cancer” program, given by two certified master trainers. Facilitators are persons with stable (advanced) cancer, cancer survivors, relatives of persons with cancer or bereaved relatives of patients who died at least six months before the facilitators’ training. A duo of the same peer facilitators facilitate all six meetings of the program. |
| 6 | HOW (modes of delivery) | The group meetings will be facilitated via the video conferencing application, “Zoom”. The groups will consist of eight to 12 participants, two peer facilitators, and one person for technical support. |
| 7 | WHERE (the type(s) of location(s) where the intervention occurred, including any necessary infrastructure or relevant features) | Each person, i.e. participants, facilitators, and the technical support person choses his/her own location. Usually, this will be at their home or at their office. |
| 8 | WHEN and HOW MUCH (the number of times the intervention was delivered and over what period of time including the number of sessions, their schedule, and their duration, intensity or dose) | The group meetings last 1,5 hours, running once a week for six weeks. To prepare for the meetings, participants will listen and watch preparatory audio clips with supportive text each week. The duration to prepare is fifteen minutes per week. Participants choose how much time they want to spend on reading the workbook. |
| 9 | TAILORING (if the intervention was planned to be personalized, titrated or adapted, then describe what, why, when, and how) | The program includes several relevant topics for the target group, covered at each meeting. Each participant decides which topic (s)he wants to work on, so the program is self-tailored. Self-tailoring can be done by setting individual goals, making individual action plans and making decisions. In addition, discussions are participant-led, ensuring they are relevant to those involved. |
