# Supplementary material for: Effectiveness of the “Living with Cancer” peer self-management support program for persons with advanced cancer and their relatives: study protocol of a non-randomized stepped wedge study
Source: BMC Palliat Care. 2022 Jun 13;21:107. doi: 10.1186/s12904-022-00994-5 (PMC9188837; doi:10.1186/s12904-022-00994-5)
Supplement: Supplementary file 3 — Additional file 3. [file 12904_2022_994_MOESM3_ESM.docx]

**List of related articles of the SMART study**

1. Van Dongen SI, De Nooijer K, Cramm JM, Francke AL, Oldenmenger WH, Korfage IJ, et al. Self-management of patients with advanced cancer: A systematic review of experiences and attitudes. Palliat Med. 2020;34(2):160-78.

2. van Dongen SI, Stoevelaar R, Kranenburg LW, Noorlandt HW, Witkamp FE, van der Rijt CCD, et al. The views of healthcare professionals on self-management of patients with advanced cancer: An interview study. Patient Educ Couns. 2022;105(1):136-44.

3. Noorlandt HW, Stoevelaar R, et al. Challenges in self-management of persons living with advanced cancer: A qualitative interview study. Eur J Cancer Care (Engl). Submitted 2022.
